# Innate Host Response in Primary Human Hepatocytes with Hepatitis C Virus Infection

**DOI:** 10.1371/journal.pone.0027552

**Published:** 2011-11-08

**Authors:** Darong Yang, Nianli Liu, Chaohui Zuo, Shoahua Lei, Xinjiao Wu, Fei Zhou, Chen Liu, Haizhen Zhu

**Affiliations:** 1 Department of Molecular Medicine, College of Biology of Hunan University, Changsha, China; 2 State Key Laboratory of Chem/Biosensesing and Chemometrics, Hunan University, Changsha, China; 3 Research Center of Cancer Prevention and Treatment of Hunan University and Hunan Provincial Tumor Hospital, Hunan Provincial Tumor Hospital, Changsha, China; 4 Department of Pathology, Immunology and Laboratory Medicine, University of Florida College of Medicine, Gainesville, Florida, United States of America; Duke University School of Medicine, United States of America

## Abstract

**Background and Aim:**

The interaction between hepatitis C virus (HCV) and innate antiviral defense systems in primary human hepatocytes is not well understood. The objective of this study is to examine how primary human hepatocytes response to HCV infection.

**Methods:**

An infectious HCV isolate JFH1 was used to infect isolated primary human hepatocytes. HCV RNA or NS5A protein in the cells was detected by real-time PCR or immunofluorescence staining respectively. Apoptosis was examined with flow cytometry. Mechanisms of HCV-induced IFN-β expression and apoptosis were determined.

**Results:**

Primary human hepatocytes were susceptible to JFH1 virus and released infectious virus. IFN-α inhibited viral RNA replication in the cells. IFN-β and interferon-stimulated genes were induced in the cells during acute infection. HCV infection induced apoptosis of primary human hepatocytes through the TRAIL-mediated pathway. Silencing RIG-I expression in primary human hepatocytes inhibited IFN-β and TRAIL expression and blocked apoptosis of the cells, which facilitated viral RNA replication in the cells. Moreover, HCV NS34A protein inhibited viral induced IFN-β expression in primary human hepatocytes.

**Conclusion:**

Innate host response is intact in HCV-infected primary human hepatocytes. RIG-I plays a key role in the induction of IFN and TRAIL by viruses and apoptosis of primary human hepatocytes via activation of the TRAIL-mediated pathway. HCV NS34A protein appears to be capable of disrupting the innate antiviral host responses in primary human hepatocytes. Our study provides a novel mechanism by which primary human hepatocytes respond to natural HCV infection.

## Introduction

Hepatitis C virus (HCV) infection is a rapidly increasing public health problem, with an estimated 170 million infected patients worldwide [1]. It causes significant liver disease ranging from chronic hepatitis to cirrhosis and even hepatocellular carcinoma [2]. Unfortunately, there is no vaccine available for HCV and only a subset of HCV patients response to interferon alpha (IFN-α) and Ribavirin treatment. In contrast to most other viral infection, the hallmark of HCV infection is that the majority patients (up to 80%) will develop chronic infection after viral exposure [1], [2]. However, around 25% to 50% of HCV infected patients resolve acute HCV infection without treatment [1], indicating that innate and/or adaptive immune responses are capable of controlling the outcome of HCV infection. Processes that regulate innate intracellular antiviral responses may therefore serve as pivotal points of control, potentially limiting host permissiveness for HCV replication.

Virus-induced production of type I IFNs and the subsequent expression of IFN-stimulated genes (ISGs) are central to these antiviral defenses [3]. Production of type I IFN by virus-infected cells is the central event in their antiviral immune response. In mammalian cells, IFN gene transcription is induced through distinct signaling pathways by viral infection or by double-strand RNA (dsRNA) treatment. IFN triggers the innate cellular antiviral response, which functions to limit viral replication.

Previous HCV infection studies have involved infected patients [4], [5] and chimpanzees [6], [7]. The recent development of infectious HCV clone (JFH1) provides a very powerful tool for the analysis of host-virus interactions [8]–[10]. However, antiviral responses have been studied in human hepatic-derived cell lines especially Huh7 and its derivative Huh7.5 cells which differ from the primary human hepatocytes (PHH) in their antiviral signaling pathways in response to viral infection [11], [12].

The current study was to explore the innate immune response of PHH to infectious HCV infection. Our study shows that infectious HCV can induce innate immune responses in PHH via induction of IFN and triggering apoptosis of infected cells. Retinoic acid inducible gene-I (RIG-I) plays a key role in HCV-induced IFN expression and regulates the induction of tumor necrosis factor-related apoptosis inducing ligand (TRAIL) and apoptosis in PHH by viral infection. Moreover, HCV NS34A protein appears to be capable of disrupting the innate antiviral host responses in primary human hepatocytes.

## Materials and Methods

### Ethics Statement

All patients signed consent forms to acknowledge participation in this study approved by the review board of the Hunan Provincial Tumor Hospital, Changsha, China. All procedures followed were in accordance with the ethical commission of Hunan Provincial Tumor Hospital, Changsha, China. Primary human hepatocytes were obtained from healthy peritumoral liver resection specimens from 13 non-HBV, non-HCV and non-HIV infected patients undergoing partial hepatectomy for primary hepatocellular carcinoma or secondary metastatic lesions due to other cancers.

### Cell culture, reagent

Primary human hepatocytes were isolated from healthy peritumoral liver tissues and cultured as described previously [13]. TRAIL or RIG-I shRNA lentiviral particles targeted human TRAIL or RIG-I was purchased from Santa Cruz Biotechnology (Santa Cruz, CA, USA). Huh7.5 cells were kindly provided by Dr. Charles Rice (Rockefeller University, New York, NY).

### Transduction of primary human hepatocytes

5×10^5^ of PHHs were plated in each well of 6-well plate and incubated overnight with TRAIL, RIG-I or control shRNA lentiviral particles at multiplicity of infection (MOI) of 1 with 5 *µ*g/mL polybrene (Santa Cruz Biotechnology, Santa Cruz, CA, USA). Culture medium was changed and cells were maintained for 48 hours prior to HCV infection.

### Transfection of primary human hepatocytes

To obtain the high efficient transfection of primary human hepatocytes, we performed the transfection experiments using Effectene transfection reagent (Qiagen, Valencia, CA). In briefly, plasmid pTOPO or pTOPO-NS34A was first mixed with Enhancer and a buffer that provides optimal salt conditions for efficient DNA condensation at room temperature for 5 minutes. Effectene Reagent was then added and the mixture was incubated for 10 minutes to allow Effectene–DNA complexes to form. The complexes were mixed with culture medium and added directly to the cells. The cells were incubated until harvested.

### Real-time PCR assays

Total cellular RNA was extracted using TRIzol (Invitrogen, Carlsbad, CA) according to the manufacturer's protocol. The primers targeted HCV, IFN-β, TRAIL, DR4, DR5 and G1P3 have been reported previously [14] and real-time PCR were performed as described previously [14]. Results were analyzed using 2.0 software (Applied Biosystem, Foster City, CA).

### Immunofluorescence

Primary human hepatocytes were seeded on glass coverslips pre-coated with rat tail collagen type I and fixed with ice-cold acetone for 10 minutes at −20°C. The cells were washed with PBS, blocked with 1∶50 goat serum for 30 minutes at room temperature and then incubated for 1 hour with mouse monoclonal anti-NS5A antibody generated at University of Florida (Gainesville, FL). The cells were stained with FITC-labeled goat anti-mouse IgG for 45 minutes at room temperature. The coverslips were extensively washed and the nuclei were counterstained with DAPI (Vector Laboratories Inc, Burlingame, CA). Fluorescent images were obtained using fluorescent microscope (Olympus, Japan).

### Flow cytometry analysis

The Annexin V-FITC apoptosis detection kit was purchased from BD Pharmingen (San Diego, CA). The procedure has been reported previously [14]. Samples were analyzed on a FACS Caliber Cytometer (BD Pharmingen, San Diego, CA). The data were analyzed with CellQuest software.

### Statistical Analysis

Student *t*-test was applied to determine statistical significance. *P* values of <0.05 were considered statistically significant.

## Results

### HCV infects PHH and induces IFN-β and ISGs in the cells

**Figure 1:**
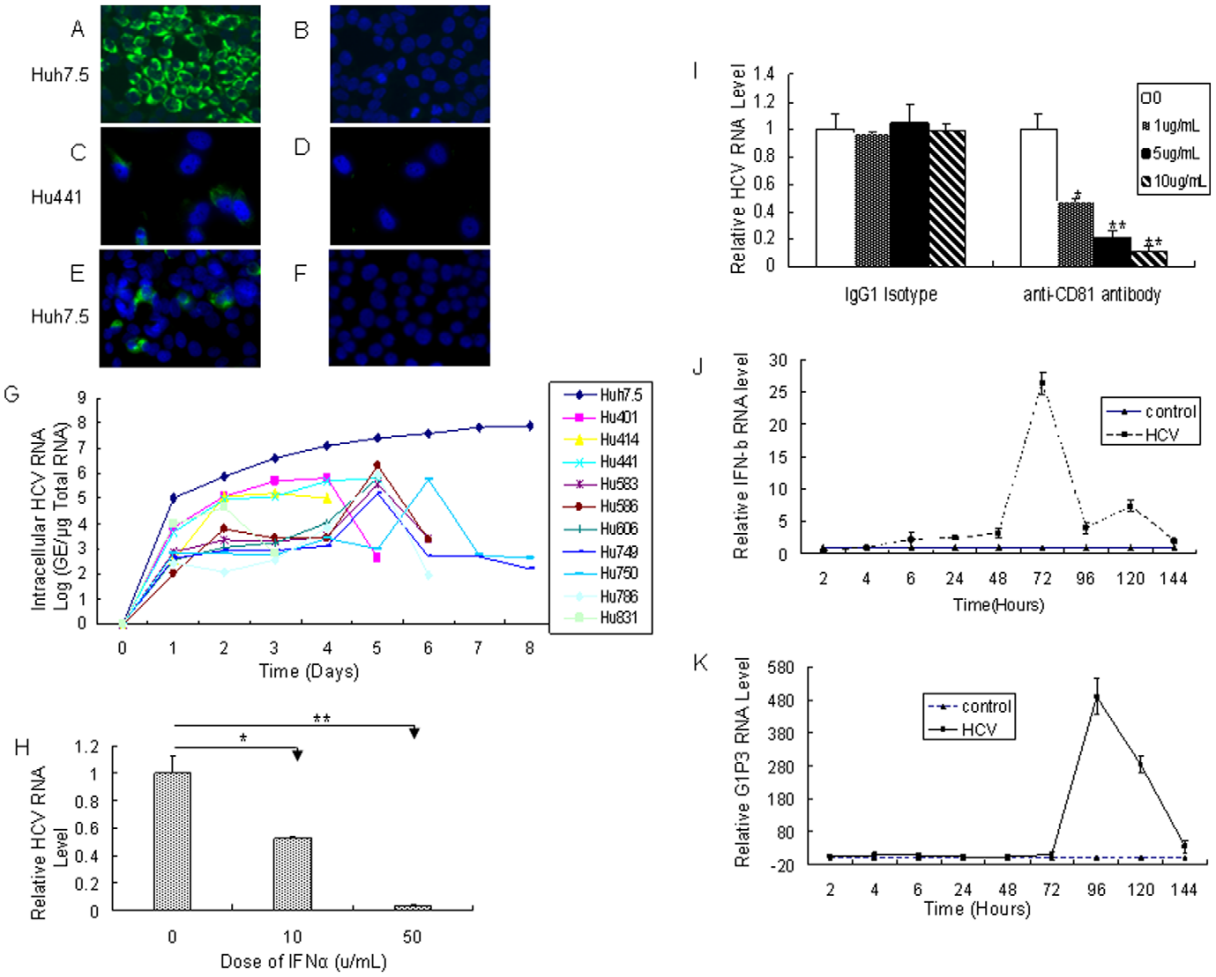
HCV infects primary human hepatocytes and induces IFN-β and ISGs in the cells. (**A**) Filtered supernatant from JFH1 RNA-transfected Huh7.5 cells was inoculated with naive Huh7.5 cells. Cells were immunostained with mouse monoclonal anti-NS5A antibody at day 5 after inoculation. DAPI was used for nuclear counterstaining. (**B**) Filtered supernatant from JFH1/GND RNA-transfected Huh7.5 cells was inoculated with naive Huh7.5 cells. Cells were stained at day 5 after inoculation as described in part A. (**C**) Filtered supernatant from JFH1 RNA-transfected Huh7.5 cells was inoculated with primary human hepatocytes Hu441. Cells were stained at day 5 after inoculation as described in part A. (**D**) Filtered supernatant from JFH1/GND RNA-transfected Huh7.5 cells was inoculated with primary human hepatocytes Hu441. At day 5 after inoculation, cells were stained as described in part A. (**E/F**) Naïve Huh7.5 cells were incubated for 3 days with filtered, conditioned media from Hu441 cells preinfection with supernatant from JFH1 RNA-transfected Huh7.5 cells (E) or Hu441 pre-inoculated with supernatant from JFH1/GND RNA-transfected Huh7.5 (F) and immunostained for NS5A expression. (**G**) Viral RNA kinetics determined by real-time PCR analysis in ten different PHH preparations and Huh7.5 cells infected by JFH1 virus. The HCV RNA in PHH was determined by quantitative real-time PCR analysis. The viral replication is represented by HCV genome equivalence (GE)/µg total RNA of HCV-infected PHH. The ten PHH preparations are primary human hepatocytes Hu401 (from 31-year-old female with liver mass in left lobe), Hu414 (from 65-year-old male with liver mass), Hu441 (from 65-year-old male with metastasis of a colic tumor), Hu583 (from 40-year-old female with liver adenoma), Hu0586 (from 69-year-old female with metastatic endometrial uterine cancer), Hu606 (from 44-year-old female with hemangioma), Hu749 (from 79-year-old female with metastatic ovarian), Hu750 (from 39-year-old female with hemangioma), Hu786 (from 70-year-old male with hepatocellular carcinoma), Hu831 (from 41-year-old male with hepatocellular carcinoma). (**H**) IFN inhibited HCV RNA replication in PHH in a dose-dependent manner. The data represent the relative RNA levels after 2 days of IFN-α treatment. These results are representative of observations made with PHH cultures from three different donors (PHHs are Hu750, Hu786 and Hu787 from 48-year-old female with metastasis of a colic tumor). (**I**) Anti-CD81 antibody blocked HCV infection in PHH. Primary human hepatocytes were pretreated with anti-CD81 antibody for 2 hours before viral inoculation. Viral RNA was analyzed by real-time PCR at day 3 post-infection. Data are means from three independent assays. These results are representative of observations made with PHH cultures from three different donors (PHH are Hu750, Hu786 and Hu787). (**J/K**) Kinetics of IFN-β and G1P3 in viral infected PHH. Three different PHH preparations (Hu583, Hu586 and Hu786) from three donors were infected with JFH1 virus at MOI of 0.1. Cells were harvested for total RNA extraction at different time points. The kinetics of induction of IFN-β (J) and G1P3 (K) was analyzed by real-time PCR assay. The data were normalized with internal control glyceraldehyde-3- phosphate dehydrogenase (GAPDH) and represent the means of 3 different PHHs from 3 different donors.

JFH1 virus, a genotype 2 virus, was cloned from a Japanese patient with fulminate hepatitis C [8]–[10]. The infection of hepatoma cell Huh7.5 by JFH1 provides an opportunity to explore the interaction between human hepatocytes and HCV. To get enough infectious HCV, we transfected viral RNA transcribed from the full-length HCV genotype 2a JFH1 or a replication-incompetent mutant (JFH1/GND) plasmid into Huh7.5 cells. Transfected cells were passaged every 3–5 days. Cell supernatant was harvested at day 18 after transfection and then used to infect naïve Huh7.5 cells. The immunofluorescence staining data showed that around 70–80% of the cells were positive for HCV NS5A protein at day 5 after viral infection (Figure 1A), while no NS5A-positive stained Huh7.5 cells inoculated with supernatant from JFH1/GND RNA-transfected cells were observed (Figure 1B). These results suggest that Huh7.5 cells transfected with JFH1 RNA are able to produce infectious viral particles.

Huh7.5 cells are poorly differentiated hepatoma cells. Moreover, they lack a functional RIG-I signaling pathway because of mutant RIG-I (T55I) in the cells and do not appear to produce detectable type I IFN when JFH1 virus is used for viral infection [14]–[16]. Thus, Huh7.5 cells may not mount an intact innate antiviral system. To better understand the interaction between the virus and its natural host cells, we isolated PHH from the human liver tissues and tried to replicate the JFH1 virus in the cells. To test whether JFH1 viruses infect PHH, we inoculated primary human hepatocytes Hu441 with the supernatant collected from JFH1 or JFH1/GND RNA-transfected Huh7.5 respectively. The cells grew on glass coverslips and were stained by immunofluorescence for HCV protein assay. As shown in Figure 1C, HCV NS5A-positive signal was only detected in Hu441 inoculated with supernatant from JFH1 RNA-transfected Huh7.5 cells, while cells incubated with supernatant from JFH1/GND RNA-transfected Huh7.5 did not show positively for HCV NS5A (Figure 1D). We inoculated other nine primary human hepatocytes preparations with the supernatant from JFH1 or JFH1/GND RNA-transfected Huh7.5 respectively and observed the similar result as shown in Hu441. In order to determine if the JFH1 virus-infected PHHs release infectious virus, we inoculated naïve Huh7.5 cells with the filtered supernatant from viral infected Hu441. Huh7.5 cells grew on the glass coverslips and were stained by immunofluorescence for HCV NS5A protein assay. As shown in Figure 1E, Huh7.5 cells inoculation with the supernatant from JFH1-infected PHH were positive for HCV NS5A protein, while cells inoculated with supernatant from JFH1/GND-pretreated PHHs did not show positively for HCV NS5A (Figure 1F). These results clearly show that JFH1-infected PHH can release infectious virus.

To get the HCV infection kinetics in PHHs, we inoculated ten different primary human hepatocyte preparations with JFH1 viruses at MOI of 0.1. Total cellular RNA was purified from the PHH at different time point. Viral RNA replication was detected with real-time PCR analysis as reported previously [14]. Viral RNA could be easily detected in ten different PHH preparations infected by JFH1 virus at different time points although it was much lower in PHH than that in Huh7.5 cells (Figure 1G). To tell the difference between the inputted viral RNA and newly synthesized viral RNA, we treated the virus-infected PHH with IFN-α or anti-CD81 antibody respectively. As shown in Figure 1H, viral RNA replication in PHH was suppressed by IFN-α in a dose-dependent manner. When PHH were treated with anti-CD81 antibody before viral inoculation, viral infection efficiency was markedly decreased in a dose-dependent manner (Figure 1I). Our data strongly indicate that primary human hepatocytes are susceptible to JFH1 virus and release infectious virus.

Production of type I IFN by virus-infected cells is the central event in their antiviral immune response. To determine whether HCV can induce IFN expression in PHHs, we infected three different PHH preparations with JFH1 virus and monitored IFN-β expression at different time points using real-time PCR assay. The expression of IFN-β in PHH was detected as early as 6 hours and reached a peak at 72 hours postinfection (Figure 1J). The protein levels in the supernatant from HCV-infected PHH were too low to be detected. The induction of IFN-β by JFH1 in Huh7.5 cells was barely detectable in similar condition.

IFN triggers the innate cellular antiviral response to limit viral replication and functions through activation of interferon stimulated genes (ISGs). Our previous study showed that G1P3 has been implicated as IFN-inducible gene [3]. G1P3 plays a functional role in IFN-induced antiviral pathway. To confirm the IFN-induced antiviral pathway is functional in PHH, we tested the expression of G1P3 in viral infected PHH. As shown in Figure 1K, the expression of G1P3 could be detected as early as 6 hours and reached a peak at 96 hours after infection and started to decrease gradually in PHH. The induction of G1P3 could not be detected in viral infected Huh7.5 cell. We tested 1-8U, another ISG, in HCV-infected PHH and observed the same phenomenon. These data strongly suggest that JFH1 virus can induce the type I IFN and ISGs in PHHs.

### HCV infection induces apoptosis of PHH through TRAIL-mediated pathway

**Figure 2:**
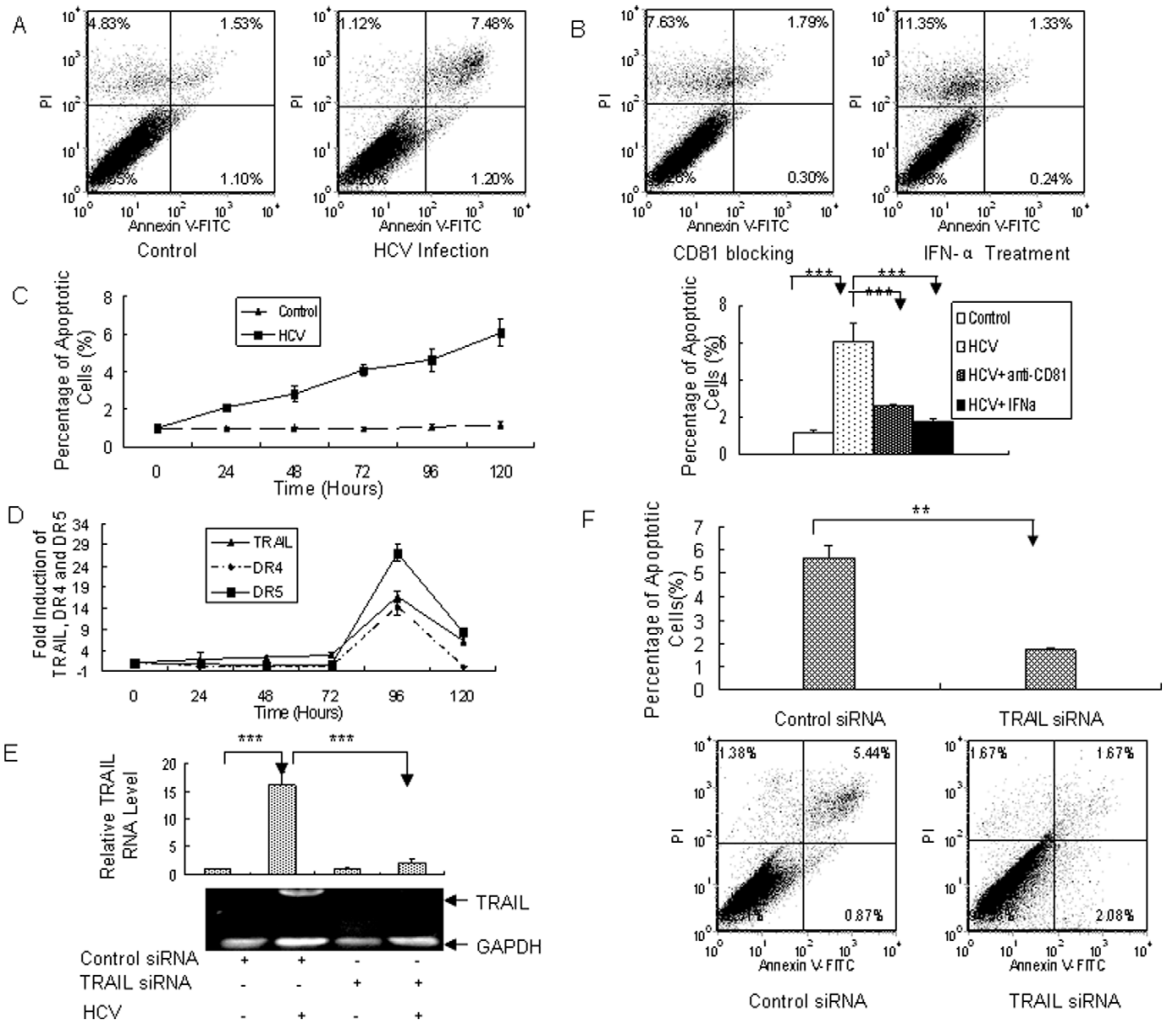
HCV induces apoptosis of PHH through TRAIL-mediated pathway. (**A**) HCV induces apoptosis of PHHs. Three different PHHs (Hu507, Hu606 and Hu831) from three donors were infected by JFH1 virus at MOI of 0.1. The cells were harvested at day 5 after infection and subjected to Annexin V analysis determined by Flow cytometry. The data represent the means of 3 different PHHs from 3 different donors. (**B**) Blocking viral entry by anti-CD81 antibody or suppression of HCV replication by IFN reduces apoptosis of PHHs. Three PHH (Hu507, Hu606 and Hu831) were treated with 100IU/mL or anti-CD81 two hours before viral infection. The cells were harvested at day 5 post-infection for Annexin V expression determined by Flow cytometry analysis. The data represent the means of 3 different PHHs from 3 different donors. (**C**) Kinetics of apoptosis in HCV-infected PHHs. Three different PHHs (Hu507, Hu606 and Hu831) from three donors were infected by JFH1 virus at MOI of 0.1. The cells were harvested every 24 hours and subjected to Annexin V analysis determined by Flow cytometry. The data represent the means of 3 different PHHs from 3 different donors. (**D**) HCV induces the expression of TRAIL, DR4 and DR5 in PHHs. Three different PHHs were infected by JFH1 virus and total RNA was purified from the cells at different time points. The expression of TRAIL, DR4 or DR5 was examined by real-time PCR analysis. The data were normalized with GAPDH and represent the means of 3 different PHHs (Hu507, Hu606 and Hu831) from 3 different donors. (**E**) TRAIL-specific siRNA knockdown HCV-induced TRAIL expression in PHH. TRAIL siRNA or control siRNA were delivered into PHH infected by HCV. The expression of TRAIL was examined using real-time PCR. The data were normalized with internal control GAPDH and represented the means of 3 different PHHs (Hu507, Hu606 and Hu831) from 3 different donors. The image showed the effectiveness of TRAIL-specific siRNA on knocking down TRAIL mRNA in HCV-infected PHHs, as determined by RT-PCR analysis using TRAIL-specific primer. (**F**) TRAIL knockdown with TRAIL-specific siRNA reduces HCV-induced apoptosis of PHHs. TRAIL siRNA or control siRNA was delivered into PHHs followed by HCV infection for 5 days. Apoptosis of PHHs was examined using flow cytometry. The data represent the means of 3 different PHHs (Hu507, Hu606 and Hu831) from 3 different donors.

Some of the primary human hepatocytes infected with JFH1 virus died, although Huh7.5 cells did not show apparent cell death after similar viral infection. To test whether cell death involves apoptosis in HCV-infected PHH, we infected three different PHH preparations with JFH1 virus and performed Annexin V staining with flow cytometry. As shown in Figure 2A, higher level of Annexin V was detected in JFH1-infected PHH compared with the control. Huh7.5 cells did not show apparent Annexin V staining after viral infection. These data indicate that JFH1 causes apoptosis in PHH. To further test that apoptosis of PHH is directly related to viral infection, we performed a series of experiments. CD81 is a critical component of HCV receptor [17], so we blocked viral infection using the antibody against human CD81 (Figure 1I). As shown in Figure 2B, blocking viral infection with anti-CD81 antibody markedly reduced apoptosis of viral infected PHH. IFN-α was used to inhibit viral replication in PPH (Figure 1H). Inhibition of HCV replication also prevented PHH from apoptosis (Figure 2B). Moreover, apoptosis was in time-dependent manner, corresponding to the viral replication in PHH (Figure 2C). These data clearly indicate that HCV infection is responsible for the apoptosis of PHH.

The activation of TRAIL death pathway has been shown in other viral infection [14], [18]. To explore the mechanisms by which HCV induces apoptosis of PHH, we examined the expression of TRAIL in virus-infected PHH using real-time PCR analysis. As shown in Figure 2D, TRAIL was detected as early as 24 hours and peaked at day 4 after infection in PHH, while the expression of TRAIL in Huh7.5 cells could not be detectable. The data indicate that the expression kinetics of TRAIL was correlated with the cell apoptosis (Figure 2C and 2D). It prompted us to hypothesize that TRAIL death pathway may be involved in the apoptosis of PHH with HCV infection because a similar pathway has been suggested in other viral infection [18]. To test our hypothesis, we examined the expression of TRAIL receptors, DR4 and DR5. As shown in Figure 2D, DR4 and DR5 were both induced and peaked at day 4 after viral infection (Figure 2D). The induction of DR4 and DR5 in Huh7.5 cells was barely detectable under similar condition. These data indicate that TRAIL signaling pathway is activated by HCV infection, which may be responsible for apoptosis of PHH.

To address the causal relationship between HCV-induced apoptosis and TRAIL activation, we performed a series of experiments. TRAIL siRNA was delivered into PHH before the cells were infected by JFH1. Apoptosis of the cells was examined by flow cytometry analysis. The data showed that TRAIL-specific siRNA efficiently knocked down its mRNA in HCV-infected PHH (Figure 2E). When TRAIL siRNA was transfected into PHH followed by viral infection, HCV-induced apoptosis was significantly reduced in comparison with the control (Figure 2F). These data strongly suggest that HCV induces apoptosis of PHH through TRAIL-mediated pathway.

### Silencing RIG-I expression in primary human hepatocytes inhibits IFN-β expression and blocks apoptosis of PHH via TRAIL-mediated pathway in response to HCV infection

**Figure 3:**
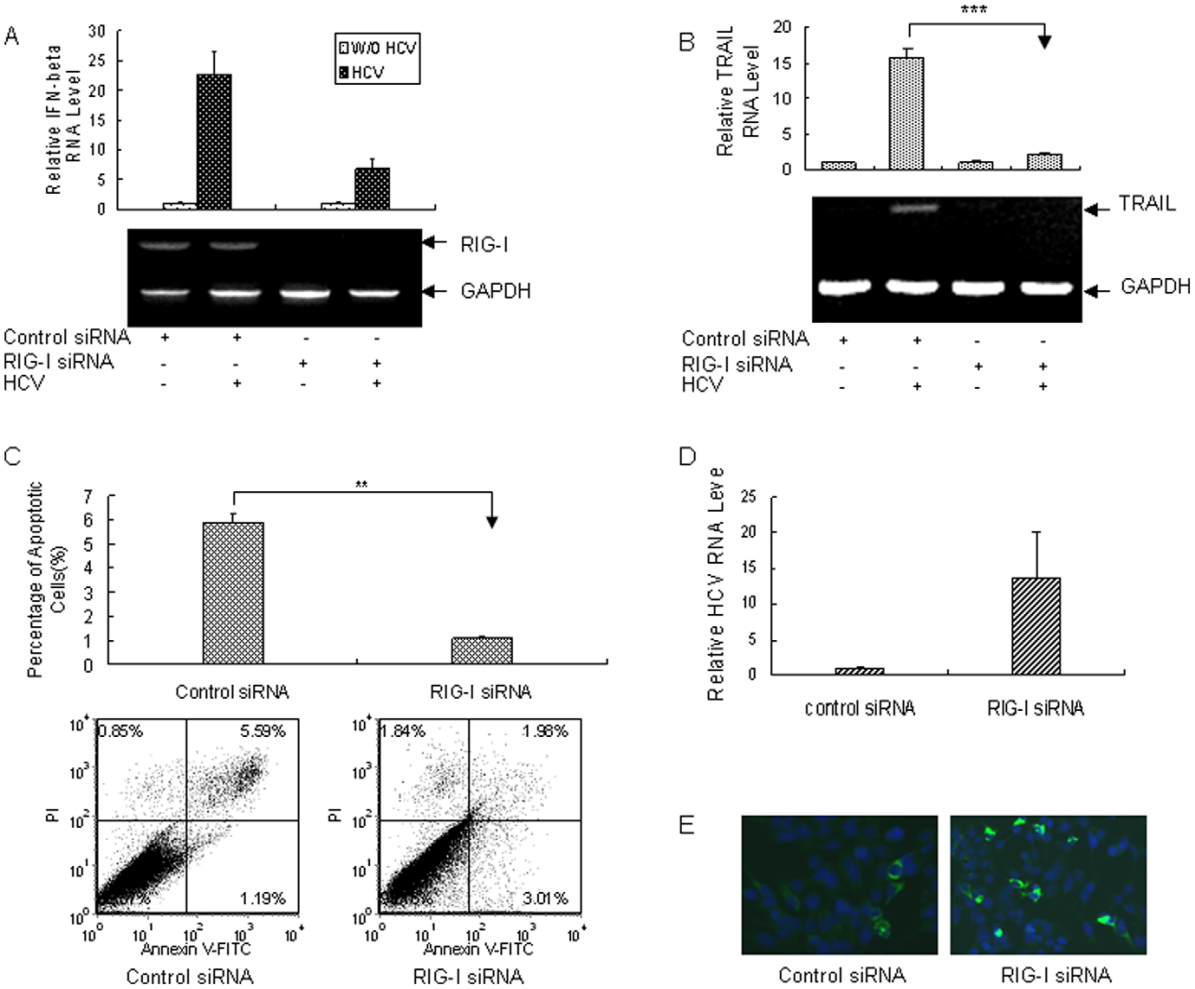
Silencing RIG-I expression in PHH inhibits the expression of IFN-β and reduces apoptosis of PHH in response to HCV infection. (**A**) RIG-I knockdown with RIG-I-specific siRNA inhibits IFN-β expression in PHH. RIG-I siRNA or control siRNA was delivered into PHH. The expression of IFN-β was examined using real-time PCR. The data were normalized with internal control GAPDH and represented the means of 3 different PHH (Hu507, Hu606 and Hu831) from 3 different donors. The image showed the effectiveness of RIG-I-specific siRNA on knocking down RIG-I mRNA in viral infected PHHs, as determined by RT-PCR analysis using RIG-I-specific primer. (**B**) RIG-I regulates TRAIL expression in HCV-infected PHH. PHHs were transfected with control siRNA or RIG-I-specific siRNA for 24 hours, followed by viral infection. The cells were harvested for total cellular RNA extraction and the expression of TRAIL in the cells was examined using real-time RT-PCR. (**C**) RIG-I knockdown with RIG-I-specific siRNA reduces HCV-induced apoptosis of PHH. RIG-I siRNA or control siRNA was delivered into PHH followed by HCV infection for 5 days. Apoptosis of PHH was examined using flow cytometry. The data represent the means of 3 different PHHs (Hu507, Hu606 and Hu831) from 3 different donors. (**D**) Relative HCV RNA level in PHH transfected with RIG-I siRNA. PHH were transfected with RIG-I or control siRNA, followed by viral infection for 72 hours. Total RNA was isolated and HCV RNA was detected using real-time PCR. The data was normalized with GAPDH and represented the means of 3 different PHHs (Hu507, Hu606 and Hu831) from 3 different donors. (**E**) Immunostaining of HCV NS5A protein in PHH with RIG-I downregulation. PHHs were transfected with RIG-I or control siRNA, followed by viral infection for 72 hours. The cells were immunostained with mouse monoclonal anti-NS5A antibody. DAPI was used for nuclear counterstaining.

It has been reported that RIG-I plays an important role in dsRNA-induced innate antiviral responses [19], [20]. To test if RIG-I plays a role in IFN induction by HCV infection in PHH, we performed siRNA knockdown experiments. We confirmed that RIG-I siRNA could significantly knock down its messenger RNA (Figure 3A). When RIG-I siRNA was delivered into PHH followed by HCV infection, IFN-β was markedly reduced in comparison with the control (Figure 3A). Moreover, knocking down RIG-I inhibited TRAIL expression in HCV-infected PHHs and significantly blocked apoptosis of the cells (Figure 3B and 3C). Inhibition of IFN-β expression and blockage of HCV-induced apoptosis caused by silencing RIG-I expression in PHH contributed to the enhanced HCV RNA replication level and elevated HCV protein level in comparison with the control (Figure 3D and 3E). All the data support that RIG-I is responsible for the induction of IFN-β and TRAIL and apoptosis of primary human hepatocytes via TRAIL-mediated pathway in response to HCV infection.

### HCV NS34A inhibits the induction of IFN-β by viral infection in primary human hepatocytes

**Figure 4:**
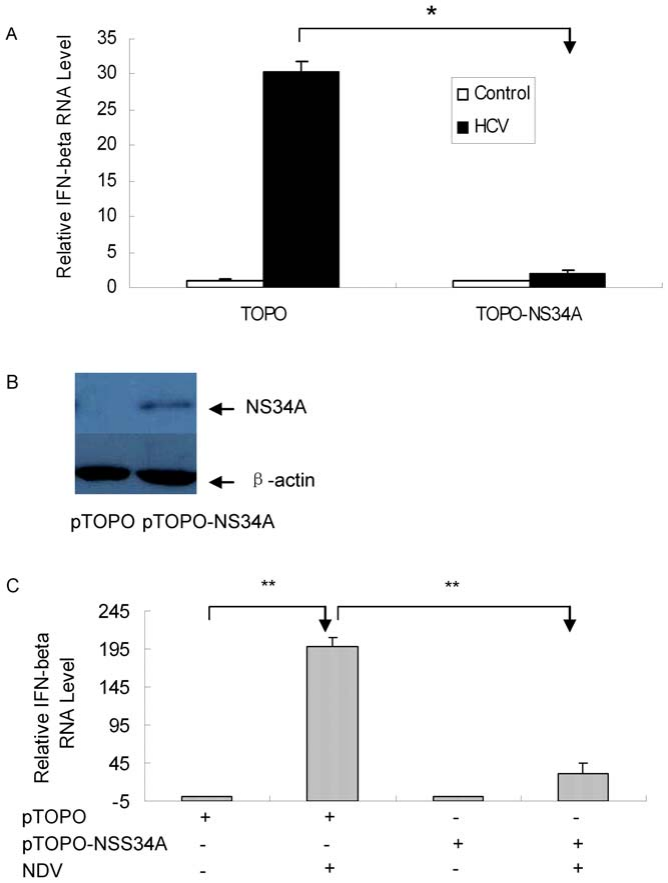
HCV NS34A inhibits IFN-β expression in viral-infected PHH. (**A**) The plasmid pTOPO-NS34A or control plasmid pTOPO was transfected into PHH, followed by HCV infection for 3 days. Total RNA was isolated and IFN-β level was detected using real-time PCR analysis. The expression level of IFN-β RNA was normalized with GAPDH. The data represent the means of 2 different PHHs (Hu507 and Hu593) from 2 different donors. (**B**) Overexpression of HCV NS34A in PHH. pTOPO or pTOPO-NS34A plasmid was transfected into PHHs respectively. Protein was isolated from the cells at day 2 after transfection and used for western blot analysis with anti- HCV NS34A antibody. (**C**) HCV NS34A inhibits the induction of IFN-β by NDV. PHH were transfected by plasmid pTOPO-NS34A or pTOPO, followed by NDV infection at MOI of 0.1. Total cellular RNA was isolated at 48 hours after infection and the level of IFN-β was determined with real-time RT-PCR analysis. The level of IFN-β RNA was normalized with GAPDH. The data represent the means of 3 different PHHs (Hu441, Hu507 and Hu593) from 3 different donors.

Many reports indicate that HCV NS34A inhibits IFN-β expression in hepatocytes [21]–[23]. However, all the studies were performed in HCV replicon cell lines or hepatoma cell transfection systems. To explore whether HCV NS34A interferes with the antiviral responses and contributes to its immune evasion strategy in PHH, we used primary human hepatocytes in our study. Our data showed that HCV replication was very low in primary human hepatocytes (Figure 1G). It may require abundant HCV NS34A protein accumulation in the PHH to execute its blockage of IFN-β induction signaling. To test our hypothesis, we overexpressed HCV NS34A protein in primary human hepatocytes to examine its impact on the induction of IFN-β by viral infection. Primary human hepatocytes were transfected with plasmid pTOPO-NS34A followed by JFH1 virus infection and the level of IFN-β was determined with real-time RT-PCR analysis. As shown in Figure 4A, the cells overexpressing HCV NS34A had much lower level of IFN-β than control cells. The expression of NS34A protein in PHH containing the plasmid pTOPO-NS34A was confirmed (Figure 4B). To further examine if HCV NS34A inhibit the induction of IFN-β by other virus, we transfected primary human hepatocytes with plasmid pTOPO-NS34A and then used Newcastle disease virus (NDV) to infect the cells. Total cellular RNA was isolated and the level of IFN-β was determined with real-time RT-PCR analysis. The data showed that HCV NS34A indeed inhibited the induction of IFN-β by NDV in PHHs (Figure 4C). All the results suggest that HCV NS34A inhibits the induction of IFN-β by viral infection in PHH.

## Discussion

Various hepatic cell models including hepatoma cell lines Huh7, Huh7.5 and immortalized human hepatocyte were used to address innate immune response to HCV. The transformed, rapidly dividing and poor differentiated hepatoma cells differ from normal primary human hepatocytes, raising doubts as to the *in vivo* relevance of the widely used in vitro system. Because HCV replication *in vivo* occurs mainly in highly differentiated, non-dividing human hepatocytes, PHH closely mimic the natural target cell of the virus and are the best model of choice for the study HCV infection physiopathology and hepatocarcinogenesis *in vitro* [24], [25]. To explore the interaction between HCV and its natural host cells, we used primary human hepatocytes in this study. Although JFH1 virus was able to infect the majority of Huh7.5 cells, only about 10% to 15% of primary human hepatocytes were infected by the same virus based on our study. In agreement with the study, one recent report showed that about 10% of hepatocytes in the liver tissue of hepatitis C patients were infected by the virus [26]. A more recent study reported production of infectious virus upon HCV inoculation of micropatterned co-cultures (MPCC) of PHH and supportive stroma in a multiwell format, but only 1-5% of the cells were infected by the virus, probably due to the microscale plate used [27]. It has been demonstrated that Huh7.5 cell line contains an inactivating mutation in RIG-I [16], an important component for interferon response via virus-related dsRNA-sensing machinery and thus lacks a functional RIG-I signaling pathway. JFH1 virus fails to induce type I IFN and ISGs expression in this cell line [14], [28], [29]. However, the same viruses are able to induce the IFN-β and ISGs expression in PHH and induction of IFN-β by viral infection in PHH depends on RIG-I signaling pathway. The levels of viral RNA were much lower in PHH than those in Huh7.5 cells. One of the reasons may be related to the activation of innate antiviral responses in PHH with HCV infection. The intact innate immune system evidenced by induction of IFN-β and ISGs in PHH and apoptosis of PHHs in response to HCV infection may contribute to viral fluctuation and low viral replication efficiency in PHH, which was demonstrated in the study.

Our data showed that cytotoxic effect of HCV infection in PHH is through TRAIL-mediated apoptosis. It has been reported that HCV infection can sensitize human hepatocytes to TRAIL-induced apoptosis [30]. Another HCV-2a strain has been reported to induce cellular apoptosis in immortalized human hepatocytes [31]. TRAIL might be a target gene of IRF-3 [32]. IRF-3 is related to TRAIL expression in viral infected hepatoma cell line [14]. The present data showed that RIG-I regulates TRAIL expression and is responsible for HCV-induced apoptosis of primary human hepatocytes via activation of TRAIL-mediated pathway. The mechanisms by which HCV induces the expression of TRAIL need to be explored. HCV core protein has been indicated in regulation of apoptosis [33]. It would be interesting to determine the impact of core protein on DR4 and DR5 expression. Our current study is the first report about that RIG-I is responsible for apoptosis of primary human hepatocytes via activation of TRAIL-mediated pathway.

Our study demonstrated the mechanism by which infectious HCV induces cytopathic and noncytopathic antiviral response in PHH. Apoptosis of HCV-infected PHH is an efficient way to eradicate viral infection. Production of type I IFN protects the uninfected cells from viral infection. It is logical for the virus to develop evasion mechanisms by which the virus minimizes its cytolytic activity and inhibits IFN production in the host cells to establish persistent infection in the presence of innate intracellular antiviral response. Many studies have provided experimental evidences to demonstrate how HCV inactivates host innate antiviral response. It has been suggested that HCV NS34A interferes with RIG-I signaling pathway and cleaves Cardif/IPS-1/VISA/MAVS, which lead to the blockage of IFN-β production [34]–[38]. However, all these studies were performed through experiments using HCV RNA replicon and hepatoma cell transfection systems. The recent development of infectious JFH1 virus provides a very powerful tool for the analysis of host-virus interactions [8]–[10]. However, antiviral responses have been studied in human hepatic-derived cell lines especially Huh7 and its derivative Huh7.5 cells which may differ from the primary human hepatocytes in their antiviral signaling pathways. It is essential to determine whether HCV NS34A interferes with the IFN-β production in primary human hepatocytes during natural viral infection. Our study demonstrated that HCV NS34A is capable of inhibiting virus-induced IFN-β expression in PHH, which facilitates persistent viral infection.

In conclusion, our study has clearly demonstrated that PHH have intact innate host response evidenced by induction of IFN-β and ISGs and apoptosis in the cells with HCV infection. RIG-I plays a key role in the induction of IFN and TRAIL and apoptosis of PHH via activation of TRAIL-mediated pathway in response to natural HCV infection. HCV NS34A protein appears to be capable of disrupting the innate antiviral host responses in PHH. These findings reveal the mechanism by which primary human hepatocytes respond to natural HCV infection. The *in vitro* system for the replication of infectious JFH1 in primary human hepatocytes should facilitate the molecular analysis of infectious virus-host interactions.
